# A Systematic Review of Cost-Effectiveness Analyses of Novel Agents in the Treatment of Multiple Myeloma

**DOI:** 10.3390/cancers13225606

**Published:** 2021-11-09

**Authors:** Maarten R. Seefat, David G. J. Cucchi, Stijn Dirven, Kaz Groen, Sonja Zweegman, Hedwig M. Blommestein

**Affiliations:** 1 Department of Hematology, Amsterdam UMC, Cancer Center Amsterdam, Vrije Universiteit Amsterdam, 1081 HV Amsterdam, The Netherlands; d.cucchi@amsterdamumc.nl (D.G.J.C.); s.dirven@student.vu.nl (S.D.); k.groen1@amsterdamumc.nl (K.G.); s.zweegman@amsterdamumc.nl (S.Z.); 2 Erasmus School of Health Policy and Management, Erasmus University Rotterdam, 3062 PA Rotterdam, The Netherlands; blommestein@eshpm.eur.nl

**Keywords:** cost-effectiveness, multiple myeloma, economic evaluation, daratumumab, carfilzomib, pomalidomide, panobinostat, elotuzumab, ixazomib

## Abstract

**Simple Summary:**

New treatments in multiple myeloma are embraced by patients and physicians but are also associated with substantial higher costs. To ensure the affordability and accessibility of health care, an evaluation of the outcomes in relation to the costs is increasingly requested. However, an up-to-date summary and assessment of the cost-effectiveness evidence for multiple myeloma treatments is currently lacking. We identified the cost-effectiveness studies currently available and show that novel treatments could improve survival with almost 4 years compared to standard of care. However, additional costs compared to standard of care could increase up to USD 535,530 per patient. The ratio between outcomes and costs is above currently accepted willingness to pay thresholds. Our results show cost-effectiveness ratios should be either improved or less favorable ratios should be accepted to ensure accessibility to promising treatments.

**Abstract:**

Background: Novel therapies for multiple myeloma (MM) promise to improve outcomes but are also associated with substantial increasing costs. Evidence regarding cost-effectiveness of novel treatments is necessary, but a comprehensive up-to-date overview of the cost-effectiveness evidence of novel treatments is currently lacking. Methods: We searched Embase, Medline via Ovid, Web of Science and EconLIT ProQuest to identify all cost-effectiveness evaluations of novel pharmacological treatment of MM reporting cost per quality-adjusted life year (QALY) and cost per life year (LY) gained since 2005. Quality and completeness of reporting was assessed using the Consolidated Health Economic Evaluation Reporting Standards. Results: We identified 13 economic evaluations, comprising 32 comparisons. Our results show that novel agents generate additional LYs (range: 0.311–3.85) and QALYs (range: 0.1–2.85) compared to backbone regimens and 0.02 to 1.10 LYs and 0.01 to 0.91 QALYs for comparisons between regimens containing two novel agents. Lifetime healthcare costs ranged from USD 60,413 to 1,434,937 per patient. The cost-effectiveness ratios per QALY gained ranged from dominating to USD 1,369,062 for novel agents compared with backbone therapies and from dominating to USD 618,018 for comparisons between novel agents. Conclusions: Cost-effectiveness ratios of novel agents were generally above current willingness-to-pay thresholds. To ensure access, cost-effectiveness should be improved or cost-effectiveness ratios above current thresholds should be accepted.

## 1. Introduction

In the last decades, the prognosis of patients with multiple myeloma (MM) improved substantially mainly due to the expanded therapeutical armamentarium [1]. Novel agents, such as the proteasome inhibitors carfilzomib and ixazomib and monoclonal antibodies, daratumumab and elotuzumab improve progression-free survival and have been introduced to standard care for (relapsed-refractory) MM patients [2,3,4,5,6]. In addition to the availability of a wealth of novel drugs, two-drug regimens used for a limited period of time are increasingly being replaced by three- to four-drug regimens used continuously until progression, which further improves survival [7]. The expected relative survival rates over five years almost doubled from 38% in 1989–2000 to 64% in 2008–2016 and are expected to rise further, since the monoclonal antibodies against CD38 (daratumumab and isatuximab) and SLAMF7 (elotuzumab) are currently also implemented in first line treatment [8,9,10].

The downside of the available novel treatments is increasing costs. In Europe, the total costs of cancer have increased from EUR 52 billion (USD 61.8 billion (calculated using the SDR per currency unit on 1 July 2021, International Monetary Fund (IMF)) [11]) in 1995 to EUR 199 billion (USD 236.5 billion (1 July 2021) [11]) in 2018. Expenditures on cancer care were EUR 103 billion (USD 122.4 billion (1 July 2021) [11]) of which almost a third was attributed to cancer medicines alone [11,12]. Rising expenditures are only in part caused by increasing incidence [13]. Compared with medicines for other indications, cancer medicines are highly priced in absolute and relative terms, and these prices are also responsible for driving up expenditures for cancer care [14]. These rising expenditures are a growing concern as they endanger affordability and accessibility to effective care for patients. Although the diagnosis of MM only accounts for a small percentage of all cancer types, the costs related to this disease are among the highest and the introduction of novel treatment options was associated with an exceptional raise in costs of MM management [15,16,17]. These were driven by costs of drug prescription, increased hospitalization and management of toxicity. Healthcare costs per patient per month among newly diagnosed MM patients in the USA shifted from USD 3,263 in 2000 to USD 14,656 in 2014 [17].

Health care decision makers increasingly require evidence regarding the cost-effectiveness of novel treatments to ensure value for money and sustainability in health care systems. Furthermore, given the numerous treatment options for MM that are available to clinicians, cost-effectiveness might be considered for guiding treatment options besides efficacy and side effects, disease and patient characteristics and previous received treatment regimens. Systematic reviews aid decisions in summarizing and assessing currently available evidence. For MM, several systematic reviews have been conducted and some of these included quality assessments [15,18,19,20,21]. However, these reviews focused on bortezomib and/or lenalidomide based regimens [19,20,21] or only included pomalidomide and carfilzomib [18]. Elotuzumab, ixazomib and panobinostat were recently reviewed. However, this review only covered results available up until 2018 [15]. A comprehensive overview of the cost-effectiveness of all novel treatment options including most recent evidence for MM is currently lacking.

In this systematic review, we sought to give a complete overview and assessment of the cost-effectiveness evidence currently available for novel treatments for MM patients. As such, we provide physicians, payers and policy makers with the necessary information for evidence-based decision making to ensure accessibility to promising novel treatment.

## 2. Methods

We conducted and report this systematic review in accordance with the Preferred Reporting Items for Systematic Reviews and Meta-Analyses (PRISMA) [22]. We submitted details of our systematic review for registration in PROSPERO (ID: 286169).

### 2.1. Eligibility Criteria

We considered all evaluations of cost-effectiveness of novel pharmacological treatment of multiple myeloma reporting an outcome of cost per QALY, cost per LY gained since 2005, when lenalidomide was approved, for inclusion. Non-English-language studies, case reports, case series, conference abstracts, studies without human subjects or MM patients, studies solely reporting list prices of drugs, out-of-pocket costs for patients or cost-of-illness were excluded. Studies reporting on cost-effectiveness of bone marrow transplantation, supportive care, prevention, palliative care, radiotherapy, surgery were excluded thereafter. At last, we only included studies with outcomes of the novel medicines daratumumab, pomalidomide, carfilzomib, elotuzumab, ixazomib and panobinostat, both monotherapy and in combination with other regimens.

### 2.2. Information Sources and Search Strategy

Embase, Medline via Ovid, Web of Science and EconLIT ProQuest were searched on the 25 February 2021. The full search strategy is available in Supplemental A. Results were de-duplicated in Endnote and imported in Rayyan (https://www.rayyan.ai/, last accessed on 29 August 2021). Two authors (M.R.S. and S.D.) independently screened all studies for eligibility, see Supplemental B for criteria. Disagreement was resolved through mutual discussion, and by arbitration by two additional authors (D.G.J.C. and H.M.B.) if necessary.

### 2.3. Data Extraction

We then extracted relevant data using a standardized data extraction form. This data extraction form included the study title and year, author, drugs of interest, total drug costs, LYs, QALYs, Incremental Cost-Effectiveness Ratio (ICER calculated as total costs per incremental LY or QALY) per LY or QALY, time horizon, mathematical model used, discount rates, perspective (e.g., payer or societal perspective), funding and country. QALYs include both quantity and quality of life and are calculated by multiplying life years by the quality of life. Utility values range from one to minus infinity. One represents perfect health and zero represents death. To calculate ICERs, the total costs of regimen A minus the costs of regimen B (incremental costs) are divided by the difference in effects of regimen A and B (incremental LYs or QALYs). ICERS were reported by their reference year and (country-specific) inflation was not implemented.

### 2.4. Quality Assessment

The quality and completeness of reporting was assessed using the Consolidated Health Economic Evaluation Reporting Standards (CHEERS) [23]. A study that scored below 14 out of 24 items was deemed to be of low reporting quality, 14–19 was moderate, and a study was of good reporting quality when scored 20 or higher [24].

### 2.5. Reporting Outcomes and Analysis

**Figure 1:**
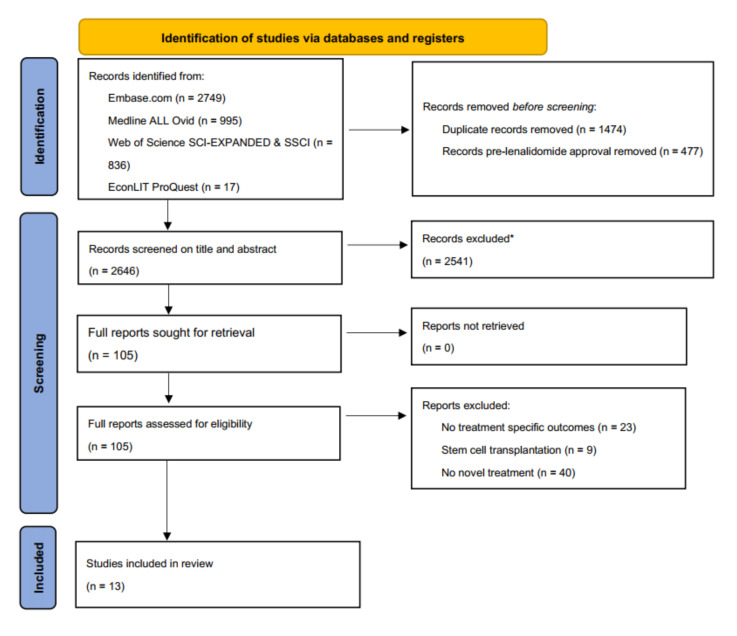
PRISMA flowchart. * Excluded on basis of exclusion criteria, e.g., no multiple myeloma, no active anti-MM treatment, no costs described, etc.

We visualized study selection with a PRISMA flowchart (Figure 1) and tabulated the characteristics and outcomes of the included studies (Table 1). To assess similarities and differences in cost-effectiveness outcomes, we converted a currency different than USD to USD using the currency rate on 1 July 2021 of the IMF [11].

## 3. Results

We identified and screened a total of 2646 single records. We excluded 2541 records based on title and abstract and reviewed full texts of 105 studies. In the final review, we included a total of 13 studies, (Figure 1). In all studies together, 32 comparisons were made, including comparisons between different lines of therapy. The comparisons included a total of 11 unique intervention regimens and eight different comparators. A summary of the included studies is presented in Table 1 [26,27,28,29,30,31,32,33,34,35,36,37,38].

### 3.1. Study Design and Structural Assumptions

Studies included in our review were published between 2016 and 2021. Eight studies (61%) were conducted in the USA [25,26,29,32,34,35,36,38], three (23%) in China [27,28,37] (of which two represented outcomes for the US context [27,28]), one in Sweden (8%) [27] and one in the Czech Republic (8%) [28]. The most chosen perspective was that of payers (nine studies) [25,26,27,28,31,34,35,36,38], followed by healthcare system (three studies) [29,32,37] and society (one study) [30]. Only one study provided evidence for the cost-effectiveness of treatments for newly diagnosed (ND)MM [38] while all other studies calculated the cost-effectiveness of treatments in relapsed or refractory (RR)MM patients [25,26,27,28,29,30,31,32,34,35,36,37].

Most studies calculated the cost-effectiveness of different regimens (e.g., addition of an additional agent to a standard regimen (backbone) or comparing two agents) [25,26,27,28,29,30,31,32,34,35,37,38]. The regimen of lenalidomide and dexamethasone (Rd) was the most frequently used comparator for calculating the incremental cost-effectiveness (i.e., for 14 comparisons (in six papers) out of 32 in total) [28,29,31,32,34,37]. One study aimed to find the most cost-effective dosing strategy of carfilzomib-dexamethasone (Kd) [36].

Effectiveness of regimens in the cost-effectiveness analyses identified by our systematic review is often derived from clinical studies. Mostly, the effectiveness was obtained from phase III RCTs [25,26,27,28,29,30,31,32,34,35,36,37], with one study using data from a meta-analysis for comparison [37] and two studies conducted an own network meta-analysis [29,33,38]. One study used three randomized phase II trials (Pelligra et al.). The source of clinical data was unclear in one study [25]. Health outcomes in terms of life years (LYs) and quality adjusted life years (QALYs) were reported in 11 and 12 studies, respectively [26,27,28,29,30,31,34,35,36,37,38]. Two studies did not report on LYs and QALYs [25,32], although detailed data for the study of Djatche et al. was available from their report by the Institute for Clinical and Economic Review (also known as “ICER”) [32,33]. Additionally, four studies reported on progression-free life years (PFLYs) and quality-adjusted progression-free life years (QAPFLYs) [32,34,35,36]. All LY and QALY outcomes are presented in Table 2.

All studies declared to have used empirical data for cost estimations (e.g., costs of adverse events, monitoring, administration and medicines), although the sources were not specified in the study of Gong et al [25,26,27,28,29,30,31,32,33,34,35,36,37,38]

A lifetime horizon was reported in 10 studies with varying definitions with a maximum of 40 years [25,27,29,30,31,32,33,34,35,36,37,38]. In contrast, Pelligra et al. used a three-year time horizon because, according to them, it reflects a typical US payer’s budget horizon and allows enough time to model clinically relevant outcomes appropriately [26]. Zhang et al. used a 10-year time horizon, without giving a rationale [28]. Most studies (10) received funding from a commercial party (e.g., pharmaceutical industry) [25,26,29,30,31,33,34,35,36,38]. All horizons and funding types are presented in Table 1.

### 3.2. Model Estimates

#### 3.2.1. Daratumumab

Three studies assessed the ICERs of the addition of daratumumab added to a backbone of Rd and/or bortezomib-dexamethasone (Vd) [27,28,29]. The ICER per QALY gained for daratumumab-Rd (DRd) versus Rd ranged from USD 187,728 to USD 1,369,062 [28,29]. The ICER per QALY gained for DVd versus Vd was USD 213,164 and USD 284,180 in two studies [27,28] One study evaluated DVd against Rd and calculated an ICER per QALY gained of USD 50,704 in second line and USD 60,359 in third line [29]. Moreover, daratumumab monotherapy was compared with pomalidomide monotherapy (Pom) for which the ICERs per QALY gained were USD 156,385 [25]. Daratumumab monotherapy was dominated by pomalidomide-dexamethasone (Pom-d) in another study [26]. All outcomes are presented in Table 2.

#### 3.2.2. Pomalidomide

Two studies evaluated the ICERs of pomalidomide. Pom-d was associated with a higher number of LYs and QALYs compared to a high dose of dexamethasone monotherapy (HiDex), though with higher costs, resulting in an ICER of USD 93,304 per QALY gained [30]. Pelligra et al. compared Pom-d to Kd and showed better outcomes at lower costs for Pom-d [26].

#### 3.2.3. Carfilzomib

In four studies, carfilzomib-Rd (KRd) was compared to Rd. The ICERs per QALY gained ranged from USD 86,938 to USD 252,293 [29,31,32,34]. Furthermore, two proteasome inhibitors were compared; carfilzomib-dexamethasone (Kd) versus bortezomib-dexamethasone (Vd), and different administration schemes of carfilzomib; high dose (70 mg/m^2^) Kd weekly (Kd70 QW) versus a lower dosage (27 mg/m^2^) of Kd twice a week (Kd27 BIW). Compared to Vd, Kd resulted in an ICER per QALY gained of USD 121,828 [35]. The total expenses of Kd70 QW were higher (USD 449,193 vs. USD 374,335), although the LYs and QALYs gained were also higher (incremental LYs 1.10, incremental QALYs 0.91), the ICER per QALY gained was calculated at USD 82,257 [36].

#### 3.2.4. Elotuzumab

Clinical data of the ELOQUENT-2 study were used in two studies evaluating the addition of elotuzumab to Rd (ERd). Calculated QALYs gained with ERd were comparable in both studies independent whether it was used as second or third line of therapy. The ICERs per QALY gained of ERd versus Rd were rather similar in the two studies and around USD 430,000 in second line and USD 480,000 in third line [29,32].

#### 3.2.5. Ixazomib

In three studies Ixazomib-Rd (IRd) was compared to Rd and in one of these studies also to Vd. Both of the studies comparing IRd with Rd used clinical data of the TOURMALINE-MM1 study and showed comparable ICERs per QALY gained (second line: USD 454,684 versus USD 433,794 and third line: USD 508,021 versus USD 484,582) [29,32]. In the third study comparing IRd with Vd and Rd, the ICERs per QALY gained were lower, USD 94,455 and USD 228,030, respectively [37].

#### 3.2.6. Panobinostat

Panobinostat in combination with Vd (Pano-Vd) dominated Rd in two studies, with lower costs and better outcomes. Incremental LYs were 1.68 and 2.02 and QALYs 1.19 and 1.42 [29,33].

### 3.3. Second vs. Third Line of Treatment

In addition to separate studies, the cost-effectiveness of KRd, IRd and ERd compared with Rd was described in a report by the Institute for Clinical and Economic Review (“ICER”). The ICERs per QALY gained of this report were presented in the article of Djatche et al. and were lower in second line versus third line [32,33]. The ICERs were also lower in second versus third line in the study of Carlson et al., comparing DVd, DRd, KRd, ERd and IRd with Rd (Table 2) [29].

### 3.4. First vs. Second Line of Treatment

**Figure 2:**
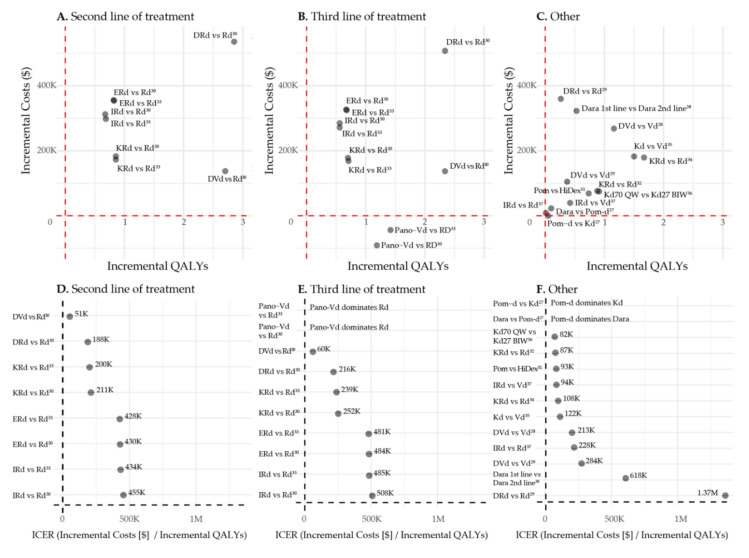
Incremental Costs, incremental QALYs and ICERs. Abbreviations: Dara: Daratumumab (monotherapy or in combination with backbone therapy), Pom: Pomalidomide monotherapy, Pom-d: Pomalidomide-dexamethasone, Kd: Carfilzomib-dexamethasone, DVd: Daratumumab-bortezomib-dexamethasone, Vd: Bortezomib-dexamethasone, DRd: Daratumumab-lenalidomide-dexamethasone, Rd: Lenalidomide-dexamethasone, KRd: Carfilzomib-lenalidomide-dexamethasone, Erd: Elotuzumab-lenalidomide-dexamethasone, Ird: Ixazomib-lenalidomide-dexamethasone, Pano-Vd: Panobinostat-bortezomib-dexamethasone, HiDex: High dose dexamethasone monotherapy, Kd70 QW: Kd 70 mg/m^2^ weekly, Kd27 BIW: Kd 27 mg/m^2^ twice per week.

Patel et al. compared daratumumab in first line with daratumumab in second line of treatment. In the used model, patients who received DRd in first line got Kd subsequently, while patients who received Rd in first line were treated with daratumumab-Kd (DKd) in second line. Lifetime healthcare costs were higher when daratumumab was used in the first line of treatment versus second line (USD 1,434,937 versus USD 1,112,101). The LYs and QALYs gained were higher over the first two lines of therapy when daratumumab was prescribed in first line (4.87 vs. 4.34 QALYs), resulting in an ICER of USD 618,018 per QALY gained [38]. All incremental costs, incremental QALYs and corresponding ICERs are depicted in Figure 2.

### 3.5. Reporting and Quality Assessment

We used the CHEERS checklist to assess quality and completeness of reporting of the studies. Most studies (12) scored well regarding reporting quality [26,27,28,29,30,31,32,33,34,35,36,37,38]. One study (Gong et al.) was of a low quality [25]. The majority of the included studies did not characterize heterogeneity (seven) [25,26,27,29,31,37,38]. Furthermore, choices for discount rates and models were not clarified in eight [25,26,28,29,30,35,36,38] and seven studies [25,26,28,29,35,37,38], respectively. All outcomes of the CHEERS checklists are presented in Table S2.

## 4. Discussion

This systematic review identified 13 economic evaluations of the cost-effectiveness of novel agents for MM, comprising a total of 11 unique intervention regimens, eight different comparators and a total of 32 comparisons. All studies were published in 2016 or later, due to our selection of novel agents (i.e., daratumumab, carfilzomib, pomalidomide, elotuzumab, ixazomib and panobinostat) [25,26,27,28,29,30,31,32,34,35,36,37,38].

Our results show that novel agents generate additional LYs ranging from 0.311 to 3.85, and additional QALYs ranging from 0.1 to 2.85 compared to backbone regimens. Comparisons between regimens containing two novel agents resulted in 0.02 to 1.10 LYs and 0.01 to 0.91 QALYs gained. This comes with high costs: lifetime healthcare costs ranging from USD 60,413 to USD 1,434,937 per patient and incremental costs compared to backbone therapies ranging from dominated to USD 535,530 per patient [25,26,27,28,29,30,31,32,34,35,36,37,38]

The ICERs we found were in only 12 (out of 32) comparisons beneath the generally accepted willingness-to-pay (WTP) threshold of USD 150,000 per QALY gained in the USA [25,26,27,28,29,30,31,32,33,34,35,36,37,38,56]. Three of these were comparisons between two novel treatment; thus, only nine comparisons were between a backbone therapy combined with a novel agent and a backbone therapy only. The European WTP thresholds in a systematic review from 2013 and later were between USD 10,196 and USD 34,097 per QALY gained [12,57]. However, higher WTP thresholds are reported by Health Technology Assessment (HTA) agencies (e.g., up to USD 95,072 in the Netherlands) [58]. Nevertheless, none of the ICERs per QALY gained (except for dominating regimens, i.e., Pano-Vd and comparisons between Pom-d and daratumumab monotherapy and Kd) fell below the WTP threshold of USD 34,097 [25,26,27,28,29,30,31,32,33,34,35,36,37,38,57]

With the WTP threshold of USD 150,000 per QALY gained taken in account, compared with backbone therapies Vd and Rd, carfilzomib and panobinostat are below the WTP in most cases [29,31,33,34,35]. The ICER per QALY gained of pomalidomide is below the WTP threshold against Kd, daratumumab monotherapy and HiDex [25,26,30]. Although daratumumab, elotuzumab and ixazomib are associated with great gains in LYs and QALYs, these medicines result in an ICER per QALY gained above USD 150,000 in most cases [25,27,28,29,32,37]. Costs are in many cases too high, making accessibility a concern. Our results show that accessibility to these novel promising medicines can only be realized if either the costs are reduced substantially, for example with price negotiations, or by accepting that the ratio between the additional benefits and the costs are above the currently known WTP thresholds (i.e., increase the WTP thresholds for MM treatments).

The regimen with the most favorable results was Pano-Vd, as this regimen dominated Rd in two studies that used clinical data of the PANORAMA-1 study [29,33]. It should be noted that Pano-Vd might not be the preferred treatment from a clinical or patient perspective, when taking other factors into account, such as adverse events [54]. The least favorable results were obtained in one study comparing DRd with Rd with an ICER of USD 1,369,062 per QALY gained, although another study conflicted with these results with ICERs per QALY gained of USD 187,728 in second line and USD 216,360 in third line with the same regimens. Although the total costs for DRd were similar in both studies, the estimated costs for Rd and the outcomes (both Lys and QALYs) show large differences. Outcomes reported by Carlson et al. were 7.38 LYs (5.44 QALYs) and 6.97 (4.38 QALYs) for DRd in second and third line, respectively, compared to 2.276 LYs (1.772 QALYs) by Zhang et al. First, the analysis by Zhang et al. was performed for RRMM patients in general while Carlson et al. focused on RRMM patients in second and third line. Second, the difference in time horizon of the analysis (i.e., 10 years for Zhang et al. 2018 versus lifetime for Carlson et al. 2018) can also explain the different outcomes. Zhang et al. (2018) base their estimates on 10 years while their model estimates show more than 30% of the patients is still alive at that time. By restricting the time horizon to 10 years, outcomes of these patients beyond 10 years are not included in their estimates [28,29].

Two studies made a total of eight comparisons between regimens in second and third line of treatment. In all cases, the incremental QALYs were higher in second line and ICERs per QALY gained were lower in second line [29,32]. One study compared daratumumab in first versus second line, in this case daratumumab in second line was more cost-effective [38].

Although most papers scored well on the CHEERS checklist, few papers characterized heterogeneity and/or provided reasons for the underlying model and discount rates. A discount rate of 3% was used in all, except one study (i.e., 92%) in our review against 71% in Asrar et al. 2020 [15,25]. Gaultney et al. reported in 2009 that only 23% of their included economic evaluations used a discount rate [21]. This implies that the reporting quality of cost-effectiveness studies is improving over time. However, only seven papers characterized heterogeneity and seven provided reasons for the underlying model [25,26,27,29,31,37,38]. This impedes comparability and we suggest these as areas for improvement for reporting future cost-effectiveness studies.

There are some limitations to this systematic review. First, we only included evidence of cost-effectiveness evidence available through peer-reviewed publications. Additional cost-effectiveness evidence is generated through national HTA bodies such as the National Institutes of Care and Excellence (NICE) in the United Kingdom [59,60]. All novel agents described in this study underwent review by NICE and the corresponding HTA reports are publicly available through the NICE web site [61]. Future studies could additionally take data from national HTA bodies into account for systematic review. Second, ICERs were reported by their reference year and (country-specific) inflation was not implemented. This possibly leads to a small underestimating of costs in older studies, although all included studies were of 2016 and later.

In the near future, some of the discussed drugs will be out of patent, for example lenalidomide in 2022 [62]. We expect that generic variants of these drugs will be sold at lower prices than the prices used in the currently identified publications [63]. The impact of lower prices for generics on the cost-effectiveness will depend on the regimens that are compared. If the price of a backbone drug is lower but present in both regimens of the comparison, the impact on the ICERs is negligible. Nevertheless, an update of our research in the future could provide more insight in the impact of generic variants on the cost-effectiveness.

All economic evaluations described estimated treatment effects based on data from RCTs. However, generalizability of findings from RCTs to the real-world population is poor [64,65]. Furthermore, all studies included in this review used drug list prices, potentially overestimating true costs for resource use. These factors might lead to overestimation of drug effectiveness and resource use, resulting in inaccurate cost-effectiveness estimates. Future studies should aim to additionally include real-world evidence, for example generated through expanded access pathways of experimental drugs [66,67,68].

## 5. Conclusions

This systematic review gives insight in the current progress in cost-effectiveness studies of the novel agents daratumumab, pomalidomide, carfilzomib, elotuzumab, ixazomib and panobinostat. We hereby set the stage for future systematic reviews for cost-effectiveness analyses reporting quality according to the CHEERs guidelines and allowing for comparisons between regimens and hopefully sequential treatment paradigms in the future.

To ensure access to novel, better treatments for MM patients now and in the future, there should be a paradigm shift toward improving cost-effectiveness. For example, by using dosing schemes with more favorable cost-effectiveness ratios, or by lowering prices with price negotiations by health care payers. If this is not possible, we should wonder whether we are on the right path with increasing costs, while WTP thresholds remain on the same level.

## Figures and Tables

**Table 1:** Characteristics of the included studies.

Study	Comparison	Time Horizon	Model	Valuta/Discount Rate	Perspective	Country	Cost Resources *	Funding
Gong et al. 2019 [25]	Dara vs. Pom	Lifetime	Markov model	Costs in 2017 USD Discount rate NA	US payer perspective	USA	Empirical data without references	Commercial
Pelligra et al. 2017 [26]	Dara vs. Pom-dPom-d vs. Kd	3 years	Economic model	Costs in 2016 USD Discount rate 3%	US payer perspective	USA	Empirical data with references	Commercial
Zeng et al. 2020 [27]	DVd vs. Vd	Lifetime	Markov model	Costs in 2018 USD Discount rate 3%	US payer perspective	China	Empirical data with references	Foundation/government grants
Zhang et al. 2018 [28]	DVd vs. Vd DRd vs. Rd	10 years	Semi-Markov model	Costs in 2017 USD Discount rate 3%	US payer perspective	China	Empirical data with references	Foundation/government grants
Carlson et al. 2018 [29]	DVd vs. Rd DRd vs. Rd KRd vs. Rd ERd vs. Rd IRd vs. Rd 2nd and 3rd line Pano-Vd vs. Rd 3rd line	Lifetime	Partition survival model	Costs in 2016 USD Discount rate 3%	US healthcare system perspective	USA	Empirical data with references	Commercial and foundation/government grants
Borg et al. 2016 [30]	Pom-d vs. HiDex	Lifetime	Economic model	Costs in 2015 SEK Discount rate 3%	Societal perspective	Sweden	Empirical data with references	Commercial
Campioni et al. 2019 [31]	KRd vs. Rd	Lifetime (40 years)	Partition survival model	Costs in 2017 Euro Discount rate 3%	Payer perspective	Czech Republic	Empirical data with references	Commercial
Djatche et al. 2018 [32]	KRd vs. Rd ERd vs. Rd IRd vs. Rd	Lifetime	Partition survival model [33] **	Costs in 2016 USD Discount rate 3%	US healthcare system perspective	USA	Empirical data with references [33] **	Report: Commercial, foundation/government grants [33] *
Jakubowiak et al. 2016 [34]	KRd vs. Rd	Lifetime (30 years)	Partition survival model, “K-GEM” model	Costs in 2015 USD Discount rate 3%	US payer perspective	USA	Empirical data with references	Commercial
Jakubowiak et al. 2017 [35]	Kd vs. Vd	Lifetime (30 years)	Partition survival model, “K-GEM” model	Costs in 2015 USD Discount rate 3%	US payer perspective	USA	Empirical data with references	Commercial
Kumar et al. 2020 [36]	Kd70 QW vs. Kd27 BIW	Lifetime (30 years)	Partition survival model, “K-GEM” model	Costs in 2018 USD Discount rate 3%	US payer perspective	USA	Empirical data with references	Commercial
Cai et al. 2019 [37]	IRd vs. Vd and Rd	Lifetime (10 years)	Markov model	Costs in 2017 USD Discount rate 3%	China’s healthcare system perspective	China	Empirical data with references	Foundation/government grants
Patel et al. 2021 [38]	Dara in 1st line vs. Dara in 2nd line	Lifetime	Markov model	Costs in 2020 USD Discount rate 3%	US payer perspective	USA	Empirical data with references	Commercial, foundation/government grants

Abbreviations: Dara: Daratumumab (monotherapy or in combination with backbone therapy (Patel et al.) [33]), Pom: Pomalidomide monotherapy, Pom-d: Pomalidomide-dexamethasone, Kd: Carfilzomib-dexamethasone, DVd: Daratumumab-bortezomib-dexamethasone, Vd: Bortezomib-dexamethasone, DRd: Daratumumab-lenalidomide-dexamethasone, Rd: Lenalidomide-dexamethasone, KRd: Carfilzomib-lenalidomide-dexamethasone, ERd: Elotuzumab-lenalidomide-dexamethasone, IRd: Ixazomib-lenalidomide-dexamethasone, Pano-Vd: Panobinostat-bortezomib-dexamethasone, HiDex: High dose dexamethasone monotherapy, Kd70 QW: Kd 70 mg/m^2^ weekly, Kd27 BIW: Kd 27 mg/m^2^ twice per week, K-GEM: Model focused on carfilzomib, USD: US Dollar, SEK: Swedisch Krona. * Outcomes are specified in Table S1. ** Reported in the report of Institute for Clinical and Economic Review (“ICER”) [33].

**Table 2:** Cost-effectiveness details of the comparisons in the included studies.

Comparison	Total Costs Regimen *	Life Years	QALYs	Cost/LY Gained	Cost/QALY Gained	Clinical Data	Study
Dara vs. Pom	NA	NA	NA	NA	USD 156,385	Prior LOTs: NA	Gong et al. 2019 [25]
Dara vs. Pom-d	Dara: USD 139,843 Pom-d: USD 130,924 Incr: USD 8,919	Dara: 1.41 Pom-d: 1.43 Incr: 0.02	Dara: 0.98 Pom-d: 0.99 Incr: 0.01	Pom-d dominates Dara (i.e., more effective and less costly)	Pom-d dominates Dara (i.e., more effective and less costly)	Median prior LOTs: 5 MM-002 and SIRIUS trials [39,40]	Pelligra et al. 2017 [26]
DVd vs. Vd	DVd: USD 399,506 Vd: USD 131,091 Incr: USD 268,415	DVd: 2.887 Vd: 1.242 Incr 1.645	DVd: 2.206 Vd: 0.947 Incr: 1.159	USD 163,184	USD 213,164	Minimally 1 prior LOT Median prior LOTs: 2 CASTOR trial [41]	Zeng et al. 2020 [27]
DVd vs. Vd	DVd: USD 462,340 Vd: USD 357,217 Incr: USD 105,123	DVd: 2.169 Vd: 1.743 Incr: 0.426	DVd: 1.655 Vd: 1.285 Incr: 0.37	USD 246,767.61	USD 284,180	Minimally 1 prior LOT Median prior LOTs: 2 CASTOR trial [41]	Zhang et al. 2018 [28]
DVd vs. Rd 2nd and 3rd line	DVd 2nd line: USD 447,182 Rd 2nd line: USD 309,997 Incr 2nd line: USD 137,185 DVd 3rd line: USD 423,119 Rd 3rd line: USD 281,754 Incr 3rd line: USD 141,365	2nd line: DVd: 7.11 Rd: 3.53 Incr: 3.58 3rd line: DVd: 6.71 Rd: 3.25 Incr: 3.46	2nd line: DVd: 5.29 Rd: 2.59 Incr: 2.70 3rd line: DVd: 4,38 Rd: 2.04 Incr: 2.34	2nd line: USD 38,320 3rd line: USD 40,857	2nd line: USD 50,704 3rd line: USD 60,359	Rd MM-009 and 010 [42,43] DVd CASTOR trial [41] Median prior LOTs: 2	Carlson et al. 2018 [29]
DRd vs. Rd 2nd and 3rd line	DRd 2nd line: USD 845,527 Rd 2nd line: USD 309,997 Incr 2nd line: USD 535,530 DRd 3rd line: USD 789,202 Rd 3rd line: USD 281,754 Incr 3rd line: USD 507,448	2nd line: DRd: 7.38 Rd: 3.53 Incr: 3.85 3rd line: DRd: 6.97 Rd: 3.25 Incr: 3.72	2nd line: DRd: 5.44 Rd: 2.59 Incr: 2.85 3rd line: DRd: 4.38 Rd: 2.04 Incr: 2.34	2nd line: USD 139,099 3rd line: USD 136,411	2nd line: USD 187,728 3rd line: USD 216,360	Rd MM-009 and 010 [42,43] DRd POLLUX trial [44] Median prior LOTs: 1	Carlson et al. 2018 [29]
DRd vs. Rd	DRd: USD 770,614 Rd: USD 410,828 Incr: USD 359,786	DRd: 2.276 Rd: 1.965 Incr: 0.311	DRd: 1.772 Rd: 1.509 Incr: 0.263	USD 1,156,868	USD 1,369,062	Minimally 1 prior LOT Median prior LOTs: 1 POLLUX trial [44]	Zhang et al. 2018 [28]
Pom-d vs. HiDex	Pom-d: USD 89,618.68 HiDex: USD 21,027.21 Incr: USD 68,591.47	Pom-d: 2.33 HiDex: 1.12 Incr: 1.21	Pom-d: 1.3904 HiDex: 0.6553 Incr: 0.7351	USD 56,687	USD 93,305	Average prior LOTs: 5 MM-003 trial [45]	Borg et al. 2016 [30]
Pom-d vs. Kd	Pom-d: USD 130,924 Kd: USD 131,119 Incr: −USD 195	Pom-d: 1.43 Kd: 1.36 Incr: 0.07	Pom-d: 0.99 Kd: 0.94 Incr: 0.05	Pom-d dominates Kd (i.e., more effective and less costly)	Pom-d dominates Kd (i.e., more effective and less costly)	Median prior LOTs: 5 MM-002 and PX-171-003-A1 trials [39,46]	Pelligra et al. 2017 [26]
KRd vs. Rd	KRd: USD 139,677.39	KRd: 3.42	KRd: 2.63	USD 77,268	USD 86,939	ASPIRE [47] (median prior LOTS: 2 (range1–3, 43.1% 1 LOT)) and RMG (Registry of Monoclonal Gammopathies)	Campioni et al. 2019 [31]
	Rd: USD 63,181.28	Rd: 2.43	Rd: 1.75				
	Incr: USD 76,496.11	Incr: 0.99	Incr: 0.88				
KRd vs. Rd 2nd and 3rd line	KRd 2nd line: USD 492,872 Rd 2nd line: USD 309,997 Incr 2nd line: USD 182,875 KRd 3rd line: USD 459,868 Rd 3rd line: USD 281,754 Incr 3rd line: USD 178,114	2nd line: KRd: 4.71 Rd: 3.53 Incr: 1.18 3rd line: KRd: 4.37 Rd: 3.25 Incr: 1.12	2nd line: KRd: 3.45 Rd: 2.59 Incr: 0.86 3rd line: KRd: 2.74 Rd: 2.04 Incr: 0.70	2nd line: USD 154,979 3rd line: USD 159,030	2nd line: USD 211,458 3rd line: USD 252,293	Rd MM-009 and 010 [42,43] KRd ASPIRE trial [47] Median prior LOTs: 2	Carlson et al. 2018 [29]
KRd vs. Rd 2nd and 3rd line	KRd 2nd line: USD 457,350 ** Rd 2nd line: USD 284,400 ** Incr: USD 172,951 ** KRd 3rd line: USD 427,027 ** Rd 3rd line: USD 258,609 ** Incr: USD 168,418 **	2nd line: KRd: 4.71 (2.34 PFLYs) ** Rd: 3.53 (1.73 PFLYs) ** Incr: 1.17 (0.61 PFLYs) ** 3rd line: KRd: 4.37 (2.12 PFLYs) ** Rd: 3.25 (1.55 PFLYs) ** Incr: 1.12 (0.57 PFLYs) **	2nd line: KRd: 3.45 (1.91 QAPFLYs) ** Rd: 2.59 (1.41 QAPFLYs) ** Incr: 0.86 (0.50 QAPFLYs) ** 3rd line: KRd 2.74 (1.37 QAPFLYs) ** Rd: 2.04 (1.00 QAPFLYs) ** Incr: 0.71 (0.37 QAPFLYs) **	2nd line: USD 147,821 ** 3rd line: USD 150,373 **	2nd line: USD 199,982 3rd line: USD 238,560	Number of prior LOTs: 1 or 2 Median prior LOTS: 2 ASPIRE trial [47]	Djatche et al. 2018 [32]
KRd vs. Rd	KRd: USD 483,845 Rd: USD 304,452 Incr: USD 179,393	KRd: 7.83 (3.79 PFLYs) Rd: 5.84 (2.59 PFLYs) Incr: 1.99 (1.20 PFLYs)	KRd: 5.88 (3.20 QAPFLYs) Rd: 4.21 (2.13 QAPFLYs) Incr: 1.67 (1.07 QAPFLYs)	USD 89,957 USD 149,834 per PFLY	USD 107,520 USD 167,379 per QAPFLY	1–3 prior LOTs Median prior LOTS: 2 ASPIRE trial [47]	Jakubowiak et al. 2016 [34]
Kd vs. Vd	Kd: USD 508,730 Vd: USD 326,032 Incr: USD 182,699	Kd: 6.59 (3.21 PFLYs) Vd: 4.93 (1.43 PFLYs) Incr: 1.66 (1.79 PFLYs)	Kd: 5.12 (2.62 QAPFLYs) Vd: 3.62 (1.13 QAPFLYs) Incr: 1.50, (1.50 QAPFLYs)	USD 109,975 USD 102,191 per PFLY	USD 121,828 USD 122,028 per QAPFLY	1–3 prior lots +/− 50% 1 prior LOT, +/50% 2–3 prior LOTs ENDEAVOR trial [48]	Jakubowiak et al. 2017 [35]
Kd70 QW vs. Kd27 BIW	Kd70 QW: USD 449,193 Kd27 BIW: USD 374,335 Incr: USD 74,858	Kd70QW: 4.17 (1.76 PFLYs) Kd27BIW: 3.07 (1.19 PFLYs) Incr: 1.10 (0.58 PFLYs)	Kd70 QW: 2.93 (1.42 QAPFLYs) Kd27 BIW: 2.02 (0.90 QAPFLYs) Incr: 0.91 (0.52 QAPFLYs)	USD 67,915 USD 129,066 per PFLY	USD 82,257 USD 143,958 per QAPFLY	2–3 prior LOTs +/− 50% 2LOTs and +/− 50% 3 LOTs ARROW trial [49]	Kumar et al. 2020 [36]
ERd vs. Rd 2nd and 3rd line	ERd 2nd line: USD 665,728 Rd 2nd line: USD 309,997 Incr: USD 355,731 ERd 3rd line: USD 608,651 Rd 3rd line: USD 281,754 Incr: USD 326,897	2nd line: ERd: 4.66 Rd: 3.53 Incr: 1.13 3rd line: ERd: 4.32 Rd: 3.25 Incr: 1.07	2nd line: ERd: 3.41 Rd: 2.59 Incr: 0.82 3rd line: ERd: 2.71 Rd: 2.04 Incr: 0.67	2nd line: USD 314,806 3rd line: USD 305,511	2nd line: USD 430,009 3rd line: USD 484,168	Rd MM-009 and 010 [42,43] ERd ELOQUENT-2 trial [50] Median prior LOTs: 2.	Carlson et al. 2018 [29]
ERd vs. Rd 2nd and 3rd line	ERd 2nd line: USD 638,144 ** Incr 2nd line: USD 353,744 ** ERd 3rd line: USD 583,531 ** Incr 3rd line: USD 324,922 **	2nd line: ERd: 4.66 (2.31 PFLYs) ** Rd: 3.53 (1.73 PFLYs) ** Incr: 1.12 (0.58 PFLYs) ** 3rd line: ERd: 4.32 (2.09 PFLYs) ** Rd: 3.25 (1.55 PFLYs) ** Incr: 1.07 (0.54 PFLYs) **	2nd line: ERd: 3.41 (1.89 QAPFLYs) ** Rd: 2.59 (1.41 QAPFLYs) ** Incr: 0.83 (0.58 QAPFLYs) ** 3rd line: ERd: 2.71 (1.36 QAPFLYs) ** Rd: 2.04 (1.00 QAPFLYs) ** Incr: 0.68 (0.35 QAPFLYs) **	2nd line: USD 315,843 ** 3rd line: USD 303,665 **	2nd line: USD 427,607 3rd line: USD 481,244	1–2 prior LOTs Median prior LOTs: 2 ELOQUENT-2 [50]	Djatche et al. 2018 [32]
IRd vs. Vd and Rd	IRd: USD 60,413 Incr: Compared with Vd: USD 39,671 Compared with Rd: USD 22,803	NA	IRd: 0.68 Incr: Compared with Vd: 0.42 Compared with Rd: 0.1	NA	Compared to Vd: USD 94,455 Compared to Rd: USD 228,030	Prior LOTs: 1 44% 2 38% 3 17% IRd vs. Rd: Hou et al. 2017 [51] Vd: Luo et al. 2018 [52]	Cai et al. 2019 [37]
IRd vs. Rd 2nd and 3rd line	IRd 2nd line: USD 622,378 Rd 2nd line: USD 309,997 Incr: USD 312,381 IRd 3rd line: USD 566,512 Rd 3rd line: USD 281,754 Incr: USD 284,758	2nd line: IRd: 4.46 Rd: 3.53 Incr: 0.93 3rd line: IRd: 4.14 Rd: 3.25 Incr: 0.89	2nd line: IRd: 3.27 Rd: 2.59 Incr: 0.68 3rd line: IRd: 2.60 Rd: 2.04 Incr: 0.56	2nd line: USD 335,894 3rd line: USD 319,953	2nd line: USD 454,684 3rd line: USD 508,021	Rd MM-009 and 010 [42,43] IRd TOURMALINE-MM1 trial [53] Median prior LOTs: 2	Carlson et al. 2018 [29]
IRd vs. Rd 2nd and 3rd line	IRd 2nd line: USD 582,428 * Incr 2nd line: USD 298,028 * IRd 3rd line: USD 530,228 * Incr 3rd line: USD 271,619 *	2nd line: IRd: 4.46 (2.21 PFLYs) * Rd: 3.53 (1.73 PFLYs) * Incr: 0.93 (0.48 PFLYs) * 3rd line: IRd: 4.14 (2.00 PFLYs) * Rd: 3.25 (1.55 PFLYs) * Incr: 0.89 (0.45 PFLYs) *	2nd line: IRd 3.27 (1.81 QAPFLYs) * Rd: 2.59 (1.41 QAPFLYs) * Incr: 0.69 (0.39 QAPFLYs) * 3rd line: IRd: 2.60 (1.30 QAPFLYs) * Rd: 2.04 (1.00 QAPFLYs) * Incr: 0.56 (0.29 QAPFLYs) *	2nd line: USD 320,460 * 3rd line: USD 305,190 *	2nd line: USD 433,794 3rd line: USD 484,582	1–2 prior LOTs TOURMALINE [53] (prior LOTs: 1 61% 2 29% 3 10%)	Djatche et al. 2018 [32]
Pano-Vd vs. Rd 3rd line	Pano-Vd 3rd line: USD 190,876 Rd 3rd line: USD 281,754 Incr: −USD 90,878	Pano-Vd:4.93 Rd: 3.25 Incr: 1.68	Pano-Vd: 3.23 Rd: 2.04 Incr: 1.19	Pano-Vd dominates Rd (i.e., more effective and less costly)	Pano-Vd dominates Rd (i.e., more effective and less costly)	Rd MM-009 and 010 [42,43] Pano-Vd PANORAMA-1 trial [54] 1 prior LOT: 51%	Carlson et al. 2018 [29]
Pano-Vd vs. Rd 3rd line	Pano-Vd: USD 196,021 ** Incr: −USD 44,084 **	Pano-Vd: 5.27 (2.59 PFLYs) ** Incr: 2.02 (1.04 PFLYs) **	Pano-Vd: 3.46 (1.82 QAPFLYs) ** Incr: 1.42 (0.82 QAPFLYs) **	Pano-Vd dominates Rd (i.e., more effective and less costly)	Pano-Vd dominates Rd (i.e., more effective and less costly)	PANORAMA-1 trial [54] 1 prior LOT: 51%	Djatche et al. 2018 [32]
Dara in 1st line vs. Dara in 2nd line	Lifetime healthcare costs when: Dara in 1st line: USD 1,434,937 Dara in 2nd line: USD 1,112,101 Incr: USD 322,836	Dara 1st line: 7.47 (total of 1st 2 lines) Dara in 2nd line: 6.80 (total of 1st 2 lines) Incr: 0.67	Dara in 1st line: 4.87 (total of 1st 2 lines) Dara in 2nd line: 4.34 (total of 1st 2 lines) Incr: 0.53	USD 481,844.78	USD 618,018	DRd: Facon et al. (MAIA) 0 prior LOTs [55] DKd: Dimopoulos et al. (CANDOR) median prior LOTs: 2 [3]	Patel et al. 2021 [38]

Abbreviations: Dara: Daratumumab (monotherapy or in combination with backbone therapy), Pom: Pomalidomide monotherapy, Pom-d: Pomalidomide-dexamethasone, Kd: Carfilzomib-dexamethasone, DVd: Daratumumab-bortezomib-dexamethasone, Vd: Bortezomib-dexamethasone, DRd: Daratumumab-lenalidomide-dexamethasone, Rd: Lenalidomide-dexamethasone, KRd: Carfilzomib-lenalidomide-dexamethasone, Erd: Elotuzumab-lenalidomide-dexamethasone, Ird: Ixazomib-lenalidomide-dexamethasone, Pano-Vd: Panobinostat-bortezomib-dexamethasone, HiDex: High dose dexamethasone monotherapy, Kd70 QW: Kd 70 mg/m^2^ weekly, Kd27 BIW: Kd 27 mg/m^2^ twice per week, NA: Not applicable, Incr: Incremental, PFLY: Progression-free Life year, QAPFLY: Quality adjusted progression-free life year, LOT: Line of treatment. * Total costs regimen stands for total costs during the time horizon as described in Table 1. ** Reported in the report of Institute for Clinical and Economic Review (“ICER”) [33].

## Supplementary Materials

The following are available online at https://www.mdpi.com/article/10.3390/cancers13225606/s1, Supplemental A: Search strategy (25 February 2021), Supplemental B: Eligibility criteria, Table S1, Cost resources, Table S2: Quality assessment, CHEERS checklist.
