# Supplementary material for: A Systematic Review of Cost-Effectiveness Analyses of Novel Agents in the Treatment of Multiple Myeloma
Source: Cancers (Basel). 2021 Nov 9;13(22):5606. doi: 10.3390/cancers13225606 (PMC8615675; doi:10.3390/cancers13225606)
Supplement: Supplementary file 1 [file cancers-13-05606-s001.zip › cancers-1380593-supplementary.pdf]

Supplemental Materials: A Systematic Review of Cost-Effectiveness Analyses of Novel Agents in the Treatment of Multiple Myeloma

Maarten R. Seefat, David G. J. Cucchi, Stijn Dirven, Kaz Groen, Sonja Zweegman and Hedwig M. Blommestein

Supplemental A, Search strategy (25 February 2021)

**Database**	**Inclusions**	**Inclusions (deduplicated)**
Embase.com	2749	2718
Medline ALL Ovid	995	183
Web of Science SCI-EXPANDED & SSCI	836	214
EconLIT ProQuest	17	8
**Total**	**4597**	**3123**

Embase.com (N=2749)

(myeloma/exp OR 'multiple myeloma cell line'/de OR (myeloma OR ((morbus OR disease*) NEAR/3 kahler)):ab,ti) AND ('economic aspect'/de OR 'cost'/exp OR 'economic decision making'/exp OR 'economic efficiency'/exp OR 'economics'/exp OR 'health economics'/de OR 'economic evaluation'/exp OR 'health care cost'/exp OR pharmacoeconomics/exp OR 'utility value'/exp OR (costs OR cost OR econom* OR pharmacoeconom*):ab,ti) NOT [conference abstract]/lim NOT ([animals]/lim NOT [humans]/lim) AND [english]/lim*

Medline ALL Ovid (N=995)

(Multiple Myeloma/ OR (myeloma OR ((morbus OR disease*) ADJ3 kahler)).ab,ti.) AND (exp Economics/ OR Economics.xs. OR (costs OR cost OR econom* OR pharmacoeconom*).ab,ti.) NOT (exp animals/ NOT humans/) AND english.la.*

Web of Science SCI-EXPANDED & SSCI (N=836)

TS=(((myeloma OR ((morbus OR disease*) NEAR/2 kahler))) AND ((costs OR cost OR econom* OR pharmacoeconom*))) AND DT=(article) AND LA=(english)*

EconLIT ProQuest (N=17)

AB,TI((myeloma OR ((morbus OR disease*) N/3 kahler)))*

Supplemental B, Eligibility criteria

Exclusion criteria:

- Non-English-language studies
- Case reports
- Case series
- Conference abstracts
- Studies without human subjects
- Studies without multiple myeloma patients
- Studies solely reporting list prices of drugs, out-of-pocket costs for patients or cost-of-illness
- Cost-effectiveness of bone marrow transplantation, supportive care, prevention, palliative care, radiotherapy or surgery

Inclusion criteria:

- An outcome of cost per quality-adjusted life year and/or cost per life year gained
- Since 2005 (when lenalidomide was approved)
- Novel medicines: Daratumumab, pomalidomide, carfilzomib, elotuzumab, ixazomib and panobinostat, both monotherapy and in combination with other regimens

Table S1. Cost resources

Study	Cost resources
Gong et al. 2019 [1]	"From literature", not specified
Pelligra et al. 2017 [2]	RED BOOK [15]<br>US centers for Medicare & Medicaid services (CMS) [16]<br>NICE [17]<br>Literature [18,19]
Zeng et al. 2020 [3]	US CMS [16]<br>Literature [4,9,20]
Zhang et al. 2018 [4]	RED BOOK [15]<br>US CMS [16]<br>Literature [9,10,21-26]
Carlson et al. 2018 [5]	RED BOOK [15]<br>US CMS [16]<br>Literature [27]
Borg et al. 2016 [6]	Swedish pharmacopeia [28]<br>Health economic evaluation of lenalidomide [29]
Campioni et al. 2019 [7]	Czech list of reimbursed medicinal products [30]<br>Czech ministry of health checklist [31]<br>List of Diagnosis related Group (DRG) codes (Czech) [32]<br>NICE [17]<br>Czech literature [33]
Djatche et al. 2018 [8]<br>"ICER report" [14]	Report [14]<br>RED BOOK [15]<br>US CMS [16]
Jakubowiak et al. 2016 [9]	RED BOOK [15], Average wholesale price if unavailable (no reference)<br>US CMS [16]<br>HCUPnet [34]<br>Amgen Inc Data retrospective study [25]
Jakubowiak et al. 2017 [10]	"Consistent with K-GEM model of Jakubowiak et al 2016" [10,34]<br>RED BOOK [15], Average wholesale price if unavailable (no reference)<br>US CMS [16]
Kumar et al. 2020 [11]	US CMS [16]<br>HCUPnet [34]<br>WAC AnalyScource [35]<br>Amgen Inc data retrospective study [25]<br>Literature [9,10]
Cai et al. 2019 [12]	China National Medical Security Administration [36]<br>Literature [37]
Patel et al. 2021 [13]	US CMS [16]<br>Memorial Sloan Kettering: DrugPricingLab [38]<br>Literature [39-41]

Table S2. Quality assessment, CHEERS checklist

CHEERS section/item	Item No	References												
		**Carlson et al. 2018 [5]**	**Gong et al. 2019 [1]**	**Pelligra et al. 2017 [2]**	**Zeng et al. 2020 [3]**	**Zhang et al. 2018 [4]**	**Borg et al. 2016 [6]**	**Campioni et al. 2019 [7]**	**Djatche et al. 2018 [8] "ICER" report [14]**	**Jakubowiak et al. 2016 [9]**	**Jakubowiak et al. 2017 [10]**	**Kumar et al. 2020 [11]**	**Cai et al. 2019 [12]**	**Patel et al. 2021 [13]**
**Title and abstract**														
Title	1	Y	Y	Y	Y	Y	Y	Y	N (P in report)	Y	Y	Y	Y	Y
Abstract	2	Y	N	Y	Y	Y	Y	Y	Y	Y	Y	Y	Y	Y
**Introduction**														
Background and objectives	3	Y	Y	Y	Y	Y	Y	Y	Y	Y	P	Y	Y	Y
**Methods**														
Target population and sub-groups	4	Y	N	Y	Y	Y	Y	Y	N (Y in report)	Y	Y	Y	Y	Y
Setting and location	5	P	Y	Y	P	Y	Y	Y	Y	Y	P	Y	Y	Y
Study perspective	6	P	Y	Y	P	Y	Y	Y	N (Y in report)	Y	Y	Y	Y	Y
Comparators	7	Y	Y	Y	Y	Y	Y	Y	Y	Y	Y	Y	Y	Y
Time horizon	8	Y	N	Y	P	P	Y	Y	N (Y in report)	Y	Y	Y	Y	Y
Discount rate	9	P	N	Y	P	P	P	Y	N (Y in report)	Y	P	P	Y	P
Choice of health outcomes	10	Y	P	P	P	P	Y	Y	P (Y in report)	Y	Y	Y	Y	Y
Measurement of effectiveness (single study-based estimates)	11a	NA	NA	NA	NA	NA	NA	NA	NA	Y	NA	NA	NA	NA
Measurement of effectiveness (synthesis-based estimates)	11b	Y	N	Y	P	Y	Y	Y	N (Y in report)	NA	P	Y	Y	Y
Measurement and valuation of preference-based outcomes	12	Y	N	Y	Y	Y	Y	Y	N (Y in report)	Y	Y	Y	Y	Y
Estimating resources and costs (single study-based economic evaluation)	13a	NA	NA	NA	NA	NA	NA	NA	NA	Y	NA	NA	NA	NA
Estimating resources and costs (model-based economic evaluation)	13b	Y	N	Y	Y	Y	Y	Y	N (Y in report)	NA	Y	Y	Y	Y
Currency, price date and conversion	14	Y	N	Y	Y	Y	Y	Y	Y	Y	Y	Y	Y	Y
Choice of model	15	P	P	Y	P	P	Y	Y	N (Y in report)	Y	P	Y	P	P
Assumptions	16	Y	N	Y	Y	Y	Y	Y	N (Y in report)	Y	Y	Y	Y	Y
Analytical methods	17	Y	P	Y	Y	Y	Y	Y	N (Y in report)	Y	P	Y	Y	Y
**Results**														
Study parameters	18	Y	Y	Y	Y	Y	Y	Y	N (Y in report)	Y	Y	Y	Y	Y
Incremental costs and outcomes	19	Y	N	Y	Y	Y	Y	Y	Y	Y	Y	Y	Y	Y

Characterizing uncertainty (single-study-based economic evaluation)	20a	NA	NA	NA	NA	NA	NA	NA	NA	Y	NA	NA	NA	NA
Characterizing uncertainty (model-based economic evaluation)	20b	Y	Y	Y	Y	Y	Y	Y	N (Y in report)	NA	Y	Y	Y	Y
Characterizing heterogeneity	21	N	N	N	Y	N	Y	N	Y	Y	Y	Y	N	N
**Discussion**														
Study findings, limitations, generalizability and current knowledge	22	Y	Y	Y	Y	Y	Y	Y	Y	Y	Y	Y	Y	Y
**Other**														
Source of funding	23	Y	Y	Y	Y	Y	Y	Y	Y	Y	Y	Y	Y	Y
Conflicts of interest	24	Y	Y	Y	Y	Y	Y	Y	Y	Y	Y	Y	N	Y
**Reporting quality and % score***		Good 87.5%	Low 47.9%	Good 93.8%	Good 85.4%	Good 87.5%	Good 97.9%	Good 95.6%	Art: Low 43.8% Rep: Good 97.9%	Good 100%	Good 87.5%	Good 97.9%	Good 89.6%	Good 91.7%

Abbreviations: Y= reported, P= partially reported, N= not reported, NA= not applicable, Art= Article (of Djatche et al.), Rep= Report (of Institute for Clinical and Economic Review ["ICER"]). *Studies were assigned 1 point per item for reported, 0.5 for partially reported and 0 for not reported. Percentage score was calculated after the exclusion of not applicable items.

References

1. Gong, C.L.; Studdert, A.L.; Liedtke, M. Daratumumab vs pomalidomide for the treatment of relapsed/refractory multiple myeloma: A cost-effectiveness analysis. *Am. J. Hematol.* **2019**, *94*, E68–E70. https://doi.org/10.1002/ajh.25372.
2. Pelligra, C.G.; Parikh, K.; Guo, S.; Chandler, C.; Mouro, J.; Abouzaid, S.; Ailawadhi, S. Cost-effectiveness of Pomalidomide, Carfilzomib, and Daratumumab for the Treatment of Patients with Heavily Pretreated Relapsed-refractory Multiple Myeloma in the United States. *Clin. Ther.* **2017**, *39*, 1986–2005.e5. https://doi.org/10.1016/j.clinthera.2017.08.010
3. Zeng, X.; Peng, L.; Peng, Y.; Tan, C.; Wan, X. Economic Evaluation of Adding Daratumumab to a Regimen of Bortezomib + Dexamethasone in Relapsed or Refractory Multiple Myeloma: Based on the Latest Updated Analysis of CASTOR. *Clin. Ther.* **2020**, *42*, 251–262.e5. https://doi.org/10.1016/j.clinthera.2019.12.007.
4. Zhang, T.T.; Wang, S.; Wan, N.; Zhang, L.; Zhang, Z.; Jiang, J. Cost-effectiveness of Daratumumab-based Triplet Therapies in Patients with Relapsed or Refractory Multiple Myeloma. *Clin. Ther.* **2018**, *40*, 1122–1139. https://doi.org/10.1016/j.clinthera.2018.05.012.
5. Carlson, J.J.; Guzauskas, G.F.; Chapman, R.H.; Synnott, P.G.; Liu, S.; Russo, E.T.; Pearson, S.D.; Brouwer, E.D.; Ollendorf, D.A. Cost-effectiveness of Drugs to Treat Relapsed/Refractory Multiple Myeloma in the United States. *J. Manag. Care Spec. Pharm.* **2018**, *24*, 29–38. https://doi.org/10.18553/jmcp.2018.24.1.29.
6. Borg, S.; Nahi, H.; Hansson, M.; Lee, D.; Elvidge, J.; Persson, U. Cost effectiveness of pomalidomide in patients with relapsed and refractory multiple myeloma in Sweden. *Acta Oncol.* **2016**, *55*, 554–560. https://doi.org/10.3109/0284186X.2015.1096021.
7. Campioni, M.; Agirrezabal, I.; Hajek, R.; Minarik, J.; Pour, L.; Spicka, I.; Gonzalez-McQuire, S.; Jandova, P.; Maisnar, V. Methodology and results of real-world cost-effectiveness of carfilzomib in combination with lenalidomide and dexamethasone in relapsed multiple myeloma using registry data. *Eur. J. Health Econ. Health Econ. Prev. Care* **2020**, *21*, 219–233. https://doi.org/10.1007/s10198-019-01122-6.
8. Djatche, L.M.; Goble, J.A.; Chun, G.; Varga, S. Evaluating Oncology Value-Based Frameworks in the U.S. Marketplace and Challenges in Real-World Application: A Multiple Myeloma Test Case. *J. Manag. Care Spec. Pharm.* **2018**, *24*, 39–46. https://doi.org/10.18553/jmcp.2018.24.1.39.
9. Jakubowiak, A.J.; Campioni, M.; Benedict, Á.; Houisse, I.; Tichy, E.; Giannopoulou, A.; Aggarwal, S.K.; Barber, B.L.; Panjabi, S. Cost-effectiveness of adding carfilzomib to lenalidomide and dexamethasone in relapsed multiple myeloma from a US perspective. *J. Med. Econ.* **2016**, *19*, 1061–1074. https://doi.org/10.1080/13696998.2016.1194278.
10. Jakubowiak, A.J.; Houisse, I.; Májer, I.; Benedict, Á.; Campioni, M.; Panjabi, S.; Ailawadhi, S. Cost-effectiveness of carfilzomib plus dexamethasone compared with bortezomib plus dexamethasone for patients with relapsed or refractory multiple myeloma in the United States. *Expert Rev. Hematol.* **2017**, *10*, 1107–1119. https://doi.org/10.1080/17474086.2017.1391088.
11. Kumar, S.K.; Majer, I.; Panjabi, S.; Medhekar, R.; Campioni, M.; Dimopoulos, M.A. Cost-effectiveness of once weekly carfilzomib 70 mg/m2 plus dexamethasone in patients with relapsed and refractory multiple myeloma in the United States. *Expert Rev. Hematol.* **2020**, *13*, 687–696. https://doi.org/10.1080/17474086.2020.1746639.
12. Cai, H.; Zhang, L.; Li, N.; Zheng, B.; Liu, M. Cost-effectiveness analysis on binary/triple therapy on the basis of ixazomib or bortezomib for refractory or relapsed multiple myeloma. *Leuk. Lymphoma* **2019**, *60*, 2951–2959. https://doi.org/10.1080/10428194.2019.1620947.
13. Patel, K.K.; Giri, S.; Parker, T.L.; Bar, N.; Neparidze, N.; Huntington, S.F. Cost-Effectiveness of First-Line Versus Second-Line Use of Daratumumab in Older, Transplant-Ineligible Patients With Multiple Myeloma. *J. Clin. Oncol. Off. J. Am. Soc. Clin. Oncol.* **2021**, *39*, 1119–1128. https://doi.org/10.1200/JCO.20.01849.
14. Ollendorf, D.A.; Chapman, R.; Khan, S.; Russo, E.T.; Synnot, P.G.; Pearson, S.D.; Carlson, J.J.; Guzauskas, G.F. Treatment Options for Relapsed or Refractory Multiple Myeloma: Effectiveness, Value, and Value-Based Price Benchmarks, Evidence Report. Institute for Clinical and Economic Review. 2016. Available online: https://icer.org/wp-content/uploads/2020/10/MWCEPAC_MM_Evidence_Report_050516-1.pdf (accessed on 10 June 2021).
15. Truven Health Analytics. RedBook. http://micromedex.com/products/product-suites/clinical-knowledge/redbook
16. United States Centers for Medicare & Medicaid Services. https://www.cms.gov/
17. National Institute for Health and Care Excellence (NICE). https://www.nice.org.uk/
18. Goulart, B.; Ramsey, S. A Trial-Based Assessment of the Cost-Utility of Bevacizumab and Chemotherapy versus Chemotherapy Alone for Advanced Non-Small Cell Lung Cancer. *Value Heal.* **2011**, *14*, 836–845, https://doi.org/10.1016/j.jval.2011.04.004.
19. Ramsey, S.D.; Clarke, L.; Kamath, T.V.; Lubeck, D. Evaluation of Erlotinib in Advanced Non-Small Cell Lung Cancer: Impact on the Budget of a U.S. Health Insurance Plan. *J. Manag. Care Pharm.* **2006**, *12*, 472–478, https://doi.org/10.18553/jmcp.2006.12.6.472.
20. Ailawadhi, S.; DerSarkissian, M.; Duh, M.S.; Lafeuille, M.-H.; Posner, G.; Ralston, S.; Zagadailov, E.; Ba-Mancini, A.; Rifkin, R. Cost Offsets in the Treatment Journeys of Patients With Relapsed/Refractory Multiple Myeloma. *Clin. Ther.* **2019**, *41*, 477–493.e7, https://doi.org/10.1016/j.clinthera.2019.01.009.
21. Jr., L.P.G.; Wang, S.; Huang, H.; Ba-Mancini, A.; Shi, H.; Chen, K.; Korves, C.; Dhawan, R.; Cakana, A.; van de Velde, H.; et al. The Cost-Effectiveness of Initial Treatment of Multiple Myeloma in the U.S. With Bortezomib Plus Melphalan and Prednisone Versus Thalidomide Plus Melphalan and Prednisone or Lenalidomide Plus Melphalan and Prednisone With Continuous Lenalidomide Maintenance Treatment. *Oncol.* **2013**, *18*, 27–36, https://doi.org/10.1634/theoncologist.2011-0380.

22. Durie, B.; Binder, G.; Pashos, C.; Khan, Z.; Hussein, M.; Borrello, I. Total cost comparison in relapsed/refractory multiple myeloma. *J. Med Econ.* **2013**, *16*, 614–622, https://doi.org/10.3111/13696998.2012.760159.
23. Janssen Biotech, Horsham, PA19044 U.S. License Number 1864. DARZALEX (daratumumab) injection, for intravenous use [prescribing information], Revision June 2017.
24. Usmani, S.Z.; Cavenagh, J.D.; Belch, A.R.; Hulin, C.; Basu, S.; White, D.; Nooka, A.; Ervin-Haynes, A.; Yiu, W.; Nagarwala, Y.; et al. Cost-effectiveness of lenalidomide plus dexamethasone vs bortezomib plus melphalan and prednisone in transplant-ineligible US patients with newly-diagnosed multiple myeloma. *J. Med Econ.* **2015**, *19*, 243–258, https://doi.org/10.3111/13696998.2015.1115407.
25. Amgen Inc. Restrospective study of healthcare utilization and costs in patients with multiple myeloma using Truven MarketScan Data Through December 2014. Thousand Oaks, CA, USA: Amgen Inc.; 2015.
26. Eber, M.R.; Laxminarayan, R.; Perencevich, E.N.; Malani, A. Clinical and Economic Outcomes Attributable to Health Care–Associated Sepsis and Pneumonia. *Arch. Intern. Med.* **2010**, *170*, 347–353, https://doi.org/10.1001/archinternmed.2009.509.
27. Roy, A.; Kish, J.K.; Bloudek, L.; Siegel, D.S.; Jagannath, S.; Globe, D.; Kuriakose, E.T.; Migliaccio-Walle, K. Estimating the Costs of Therapy in Patients with Relapsed and/or Refractory Multiple Myeloma: A Model Framework. *Am. Heal. drug benefits* **2015**, *8*, 204–215.
28. Farmaceutiska Specialiteter i Sverige [Internet]. 2013. Available from: http://www.fass.se
29. Celgene. REVLIMID (lenalidomide) – Health economic report. Celgene AB, 2007 Aug 24.
30. Státní Ústav Pro Kontrolu Léčiv: List of reimbursed medicinal products valid as of 1.12.2017. http://www.sukl.eu/sukl/list-of- reimbursed-medicinal-products-valid-as-of-1-12-2017 (2017).
31. Ministerstvo vnitra: Vyhláška č. 421/2016 Sb., kterou se mění vyhláška Ministerstva zdravotnictví č. 134/1998 Sb., kterou se vydává seznam zdravotních výkonů s bodovými hodnotami, ve znění pozdějších předpisů. https://szv.mzcr.cz/Vyhlaska
32. Ministerstvo zdravotnictví: Metodické materiály pro rok 2017. Materiály dle Sdělení CSÚ č. 313/2016 Sb., o aktualizaci Klasifi- kace hospitalizovaných (IR-DRG). http://www.mzcr.cz/Odbornik/ dokumenty/metodicke-materialy-pro-rok-2017_12858_1058_3.Html
33. Státní Ústav Pro Kontrolu Léčiv: Správní řízení. sp. zn. SUKLS58379/2014 (LP Imnovid). https://verso.sukl.cz/fcgi/verso .fpl?fname=vp_fspis (2014).
34. HCUPnet National Inpatient Sample (NIS), Agency for Healthcare Research and Quality (AHRQ). National statistics by principal diagnosis code. Rockville, MD: Agency for Healthcare Research and Quality; 2013. http://hcupnet.ahrq.gov/
35. Analysource. Wholesale acquisition cost (WAC); 2018. Available from: https://www.analysource.com/
36. China National Medical Security Bureau. Notice on the inclusion of 17 kinds of anticancer drugs in the national basic medical insurance, work injury insurance and maternity insurance drugs list. Available from: http://www.gov.cn/xinwen/2018-10/10/content_ 5328891.htm
37. Zhou, X.; Xia, J.; Mao, J.; Cheng, F.; Qian, X.; Guo, H. Real-world outcome and healthcare costs of relapsed or refractory multiple myeloma: A retrospective analysis from the Chinese experience. *Hematology* **2016**, *21*, 280–286, https://doi.org/10.1080/10245332.2015.1122259.
38. Memorial Sloan Kettering: DrugPricingLab. https://drugpricinglab.org/tools/drug-abacus/methods/
39. Patel, K.K.; Isufi, I.; Kothari, S.; Davidoff, A.J.; Gross, C.P.; Huntington, S.F. Cost-effectiveness of first-line vs third-line ibrutinib in patients with untreated chronic lymphocytic leukemia. *Blood* **2020**, *136*, 1946–1955, https://doi.org/10.1182/blood.2020004922.
40. Fiala, M.A.; Gettinger, T.; Wallace, C.L.; Vij, R.; Wildes, T.M. Cost differential associated with hospice use among older patients with multiple myeloma. *J. Geriatr. Oncol.* **2019**, *11*, 88–92, https://doi.org/10.1016/j.jgo.2019.06.010.
41. Barnes, J.I.; Divi, V.; Begaye, A.; Wong, R.; Coutre, S.; Owens, U.K.; Goldhaber-Fiebert, J.D. Cost-effectiveness of ibrutinib as first-line therapy for chronic lymphocytic leukemia in older adults without deletion 17p. *Blood Adv.* **2018**, *2*, 1946–1956, https://doi.org/10.1182/bloodadvances.2017015461.
